# MicroRNA-524 promotes cell proliferation by down-regulating PTEN expression in osteosarcoma

**DOI:** 10.1186/s12935-018-0612-1

**Published:** 2018-08-13

**Authors:** Ming Zhuang, Xubin Qiu, Dong Cheng, Chenlei Zhu, Liang Chen

**Affiliations:** 1 Department of Orthopaedic Surgery, The First Affiliated Hospital of Soochow University, No 188 Shizi Street, Suzhou, 215006 People’s Republic of China; 2 Department of Orthopaedic Surgery, The Third Affiliated Hospital of Soochow University, 185 Juqian Street, Changzhou, 213003 People’s Republic of China

**Keywords:** MicroRNA-524, PTEN, Osteosarcoma, PI3K/AKT

## Abstract

**Background:**

Increasing numbers of studies have examined the correlation between specific miRNAs and tumours to enable their diagnosis and treatment. However, there are few reports regarding the concrete role and mechanism of miRNA in osteosarcoma.

**Methods:**

The expression of miR-524 in osteosarcoma tissues and cell lines was examined by qRT-PCR. The cell proliferation was examined using CCK-8 in vitro. A series of bioinformatics and molecular biology techniques were adopted to investigate the regulatory relationship between miR-524 and target genes in osteosarcoma.

**Results:**

The results showed that the miRNA with the most significant differential expression in osteosarcoma was miR-524, which was significantly up-regulated in both osteosarcoma tissues and cell lines. MiR-524 knockdown inhibited proliferation and promoted apoptosis of osteosarcoma cells, while overexpression of miR-524 induced their proliferation. Bioinformatics analysis and luciferase assay confirmed that PTEN was a direct target gene of miR-524 and that miR-524 induced proliferation of osteosarcoma cells through activation of the PI3K/AKT pathway via inhibition of PTEN.

**Conclusions:**

MiR-524 induces the proliferation of osteosarcoma cells through activation of the PI3K/AKT pathway via inhibition of the target gene PTEN, which provides a theoretical basis for selecting a new therapeutic target for osteosarcoma.

**Electronic supplementary material:**

The online version of this article (10.1186/s12935-018-0612-1) contains supplementary material, which is available to authorized users.

## Background

Osteosarcoma is a common primary malignant tumour derived from mesenchymal tissues and can be found in patients of all ages, but it most commonly occurs in children and adolescents. Sixty percent of patients with osteosarcoma are younger than 25 years old [1]. Osteosarcoma can occur in any bone in the body, but it is most often found in the distal femur, proximal tibia and proximal humerus and often involves the metaphysis. Osteosarcoma is highly malignant, and pulmonary metastasis often precedes resection of the primary tumour. Even though tumours can be locally controlled, most patients have a poor prognosis and die from metastasis [2]. Since the 1970s, the 5-year survival rate for osteosarcoma patients has increased to approximately 70% due to comprehensive treatment from increased understanding of osteosarcoma, especially with the application of adjuvant therapies such as chemotherapy and biotherapy [3–5]. There are severe adverse effects of systemic chemotherapy, including organ damage. Moreover, surgical treatment has a serious impact on limb function, making it difficult for patients to choose this option. Therefore, identifying new and effective gene therapy targets with fewer adverse effects is key.

MicroRNA (miRNA) is a type of non-coding small RNA that is 22–28 nucleotides in length. MiRNA was recently discovered, and it is common in eukaryotes. MiRNA induces degradation or inhibits translation of target miRNAs through complete or incomplete complementary binding to the 3′-UTR of target mRNA, thereby regulating expression of target genes and affecting cell proliferation, differentiation and apoptosis. Distinct from siRNA, miRNA has multiple target genes, which, as a trigger of endogenous RNA interference, is closer to the physiological level of regulation that occurs in cells than that of a simple gene knockout [6]. MiRNA builds complex regulatory networks in the body through upstream and downstream genes and is involved in a series of important vital processes, including the pathology in human diseases. Thus, miRNA is attracting increasing attention [7]. In particular, increasing numbers of studies have examined the correlation between specific miRNAs and tumours, as well as the mechanism of miRNAs that may enable tumour diagnosis and treatment [8–11]. However, there are few reports concerning the concrete role and mechanism of miRNA in osteosarcoma in either China or foreign countries [12].

In this study, differentially expressed miRNA was screened in osteosarcoma and para-carcinoma tissues using the chip, and miRNA was subsequently screened by quantitative real-time polymerase chain reaction (qRT-PCR). These analyses pointed to miR-524, which exhibited the most significant differential expression and became the object of this study. A series of bioinformatics analyses and molecular biology techniques were adopted to investigate the regulatory relationship between miR-524 and target genes in osteosarcoma. The molecular mechanism of miR-524 in regulating the occurrence and development of osteosarcoma was further clarified to provide new interventional targets for the treatment of osteosarcoma and a theoretical basis for optimization of a treatment strategy.

## Materials and methods

### Tissue specimens

Twenty osteosarcoma specimens were collected from patients receiving surgical resection and confirmed via postoperative pathological examination at the Changzhou First People’s Hospital from 2014 to 2016. Specimens were soaked in liquid nitrogen and stored at − 80 °C. Collection of clinical specimens was approved by the Ethics Committee of Changzhou First People’s Hospital according to the Declaration of Helsinki. All enrolled patients provided informed consent.

### Differential expression of miRNA

MiRNA profiling GSE99671 was downloaded from the GEO database, which was used to investigate miRNA and mRNA expression in 18 matched pairs of osteosarcoma and para-carcinoma tissues.

### Cell culture

The osteosarcoma cell lines MG-63, 143B, Saos-2 and UMR-106 and the human normal osteoblast hFOB 1.19 line were purchased from the American Type Culture Collection (ATCC, Manassas, VA, USA). After cells were thawed, they were cultured in RPMI-1640 medium containing 10% FBS in an incubator with 5% CO_2_ at 95% humidity and 37 °C.

### Cell transfection

The PTEN overexpression plasmid and controls were purchased from Sigma-Aldrich (Shanghai, China). MiR-524 mimics, miR-524 inhibitors and negative controls were purchased from RiboBio (Guangzhou, China). The PI3K/AKT signalling pathway inhibitor LY294002 was purchased from Calbiochem (Darmstadt, Germany). Cells were cultured in RPMI-1640 medium containing 10% FBS with 5% CO_2_ at 37 °C. Cells were transfected using Lipofectamine 2000 according to manufacturer’s instructions. Transfection efficiency was assessed by fluorescence microscopy after 24 h to determine whether subsequent experiments could be performed.

### qRT-PCR

RNA was extracted from tissues and cells using TRIzol (Sigma, USA) and was subsequently reverse transcribed into cDNA using the Roche reverse transcription kit (Roche, Switzerland) followed by RT-PCR using SYBR Green Realmaster Mix kit (Tiangen, China) and ABI ViiA 7. MiRNA and mRNA expression levels were determined using the 2^−ΔΔCt^ method. GAPDH was used as the internal reference mRNA, and U6 was used as the internal reference for miRNA. All primers are shown in Additional file 1: Table S1.

### Western blotting

Forty-eight hours after transfection, total protein was extracted, followed by sodium dodecyl sulfate-polyacrylamide gel electrophoresis (SDS-PAGE). Then, protein was transferred onto a polyvinylidene fluoride (PVDF) membrane, blocked with 5% skim milk powder at room temperature for 2 h, supplemented with PTEN primary antibody diluted at 1:1000, and incubated at 4 °C overnight. The following day, the membrane was washed with PBST for 30 min, supplemented with HRP-labelled IgG antibody diluted at 1:1000, and incubated at room temperature for 2 h. The grey value of each band was detected using the Chemi Genius gel imaging system, with GAPDH as the internal reference, and the relative grey value was calculated (gray value of target band/gray value of internal reference).

### Luciferase reporter assay

Wild-type and mutant fluorescent plasmids psiCheck2-PTEN 3′-UTR and psiCheck2-PTEN 3′-UTR-MUT-Luc were constructed. MG-63 was co-transfected with miR-524 mimic, psiCheck2-PTEN 3′-UTR-WT-Luc, and psiCheck2-PTEN 3′-UTR-MUT-Luc vector, and luciferase activity in experimental and control groups was determined using the luciferase reporter assay.

### Detection of cell proliferation activity via CCK-8 assay

After transfection for 6–12 h, cells were digested in a 6-well plate, washed, centrifuged, mixed and inoculated into 96-well plates at a density of 2 × 10^3^. Six repeat wells were used for each group of cells, and six blank controls were used to remove background signals. After culture for 24, 48, 72 and 96 h, 10 μL CCK-8 reagent was added into each well and incubated for another hour. The absorbance of each well was determined at 490 nm using a microplate reader (Labsystem). After the background was removed, the cell proliferation curve was plotted, and the experiment was repeated in triplicate.

### Detection of apoptosis

A sample of cells in the logarithmic growth phase was taken, and after transfection for 48 h, single-cell suspensions at a concentration of 1 × 10^9^/L were prepared. After washing with PBS twice, 100 μL cell resuspension was combined with 5 μL Annexin V·FITC and 5 μL PI for a reaction at room temperature in the dark for 15 min. Then, 400 μL binding buffer was added to the mixture, apoptosis was assessed using flow cytometry within 1 h of reaction, and the results were averaged.

### Statistics

All data were statistically analysed using GraphPad Prism 5.0 statistical software. Data are presented as the mean ± standard deviation (x ± sd), with a one-way analysis of variance or paired t-test used to analyse differences. The Kaplan–Meier method was adopted to calculate overall survival of patients, and univariate log-rank and multivariate Cox regression analysis were used to evaluate the prognostic significance of miR-524. We considered *P *< 0.05 to indicated statistically significant differences.

## Results

### High expression of miR-524 in osteosarcoma

**Fig. 1:**
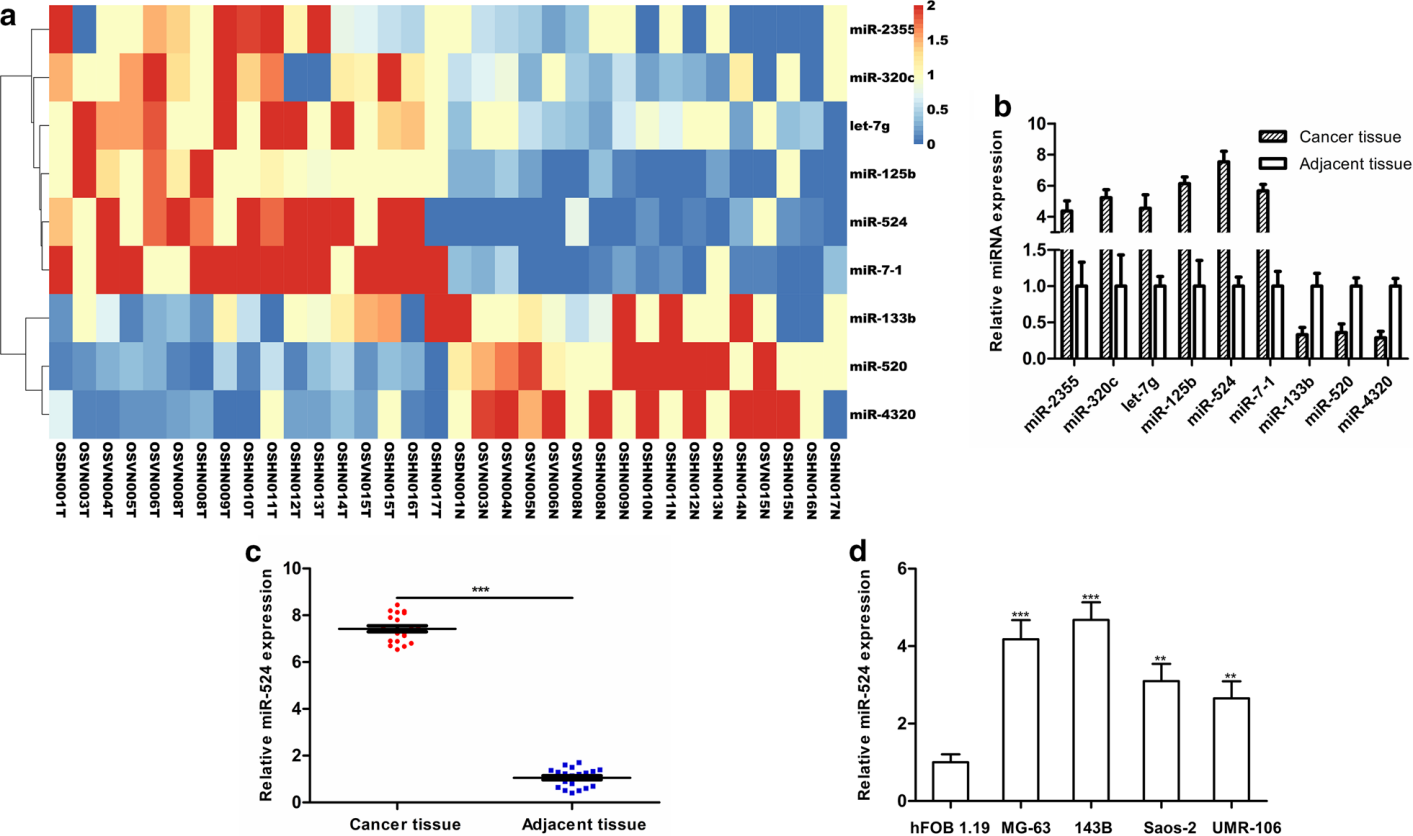
**a** Analysis of differentially expressed miRNAs in osteosarcoma based on GSE99671 (fold change ≥ 2 and *P *< 0.05). **b** qRT-PCR on nine selected miRNAs with the highest differential expression between osteosarcoma and para-carcinoma tissues. **c** MiR-524 is the most significantly up-regulated in osteosarcoma. **d** Detection of differences between miR-524 expression in normal osteoblasts (hFOB 1.19) and osteosarcoma cell lines (MG-63, 143B, Saos-2 and UMR-106) (***P < 0.001, **P < 0.01, *P < 0.05)

To study differential expression of miRNAs in osteosarcoma, sequencing data from 18 pairs of osteosarcoma and para-carcinoma tissues in GSE99671 were analysed. A total of 959 miRNAs were detected in GSE99761, and only nine miRNAs exhibited differential expression in osteosarcoma (FC ≥ 2 and *P *< 0.05), in which miR-2355, miR-320c, let-7g, miR-125b, miR-524 and miR-7-1 were highly expressed in osteosarcoma tissue, while miR-133b, miR-520 and miR-4320 exhibited reduced expression in osteosarcoma tissue (Fig. 1a). Next, we determined the differential expression of these nine miRNAs in 20 pairs of osteosarcoma and para-carcinoma tissues via qRT-PCR. We found that miR-524 was highly expressed in osteosarcoma tissues and that differential expression was the most significant in the nine miRNAs (Fig. 1b, c). Expression of miR-524 in osteosarcoma cell lines (MG-63, 143B, Saos-2 and UMR-106) and normal osteoblasts (hFOB 1.19) was measured, and we found that miR-524 was also highly expressed in osteosarcoma cells (Fig. 1d).

### MiR-524 enhances proliferation of osteosarcoma cells

**Fig. 2:**
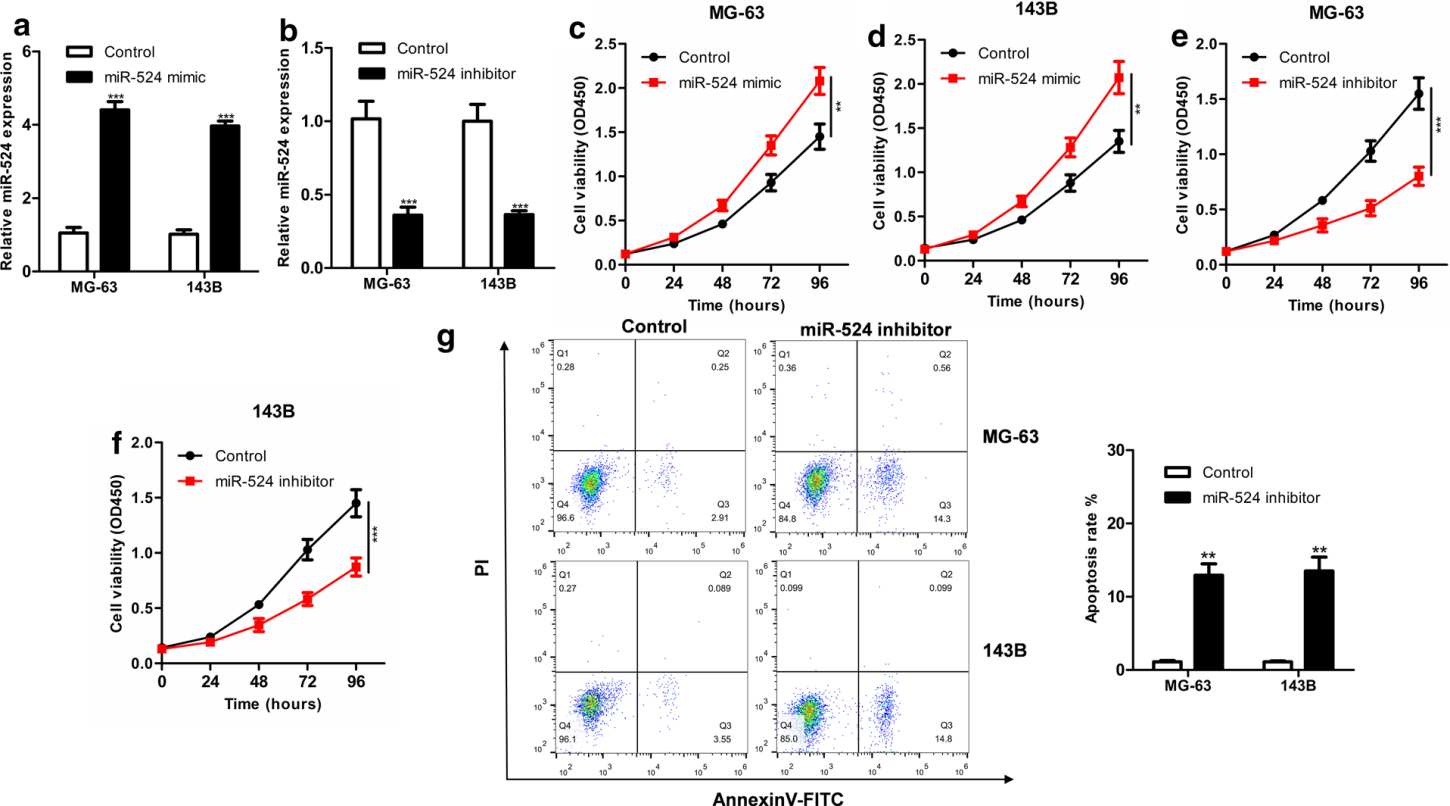
Verification efficiency of up (**a**) or down-regulated (**b**) miR-524 expression in MG-63 and 143B cell lines by qRT-PCR. After overexpression of miR-524 in MG-63 (**c**) and 143B cells (**d**), proliferation is significantly enhanced. After down-regulation of miR-524 expression in MG-63 (**e**) and 143B cells (**f**), proliferation is significantly decreased. **g** After down-regulation of miR-524 expression in MG-63 and 143B cells, apoptosis is significantly increased (***P < 0.001, **P < 0.01, *P < 0.05)

We found that miR-524 was highly expressed in MG-63 and 143B compared to that in two osteosarcoma cell lines, so these two cell lines were selected for subsequent studies. First, expression of miR-524 in MG-63 and 143B cells transfected with miR-524 mimic or miR-524 inhibitor was measured via qRT-PCR (Fig. 2a, b). Next, CCK8 proliferation assay revealed that overexpression of miR-524 significantly promoted proliferation of MG-63 and 143B cells (Fig. 2c, d), while knockdown of miR-524 significantly inhibited proliferation in both cell lines (Fig. 2e, f). In addition, apoptosis was significantly promoted in MG-63 and 143B cells in response to down-regulation of miR-524 expression compared to that in controls (Fig. 2g).

### Screening and validation of miR-524 target genes

**Fig. 3:**
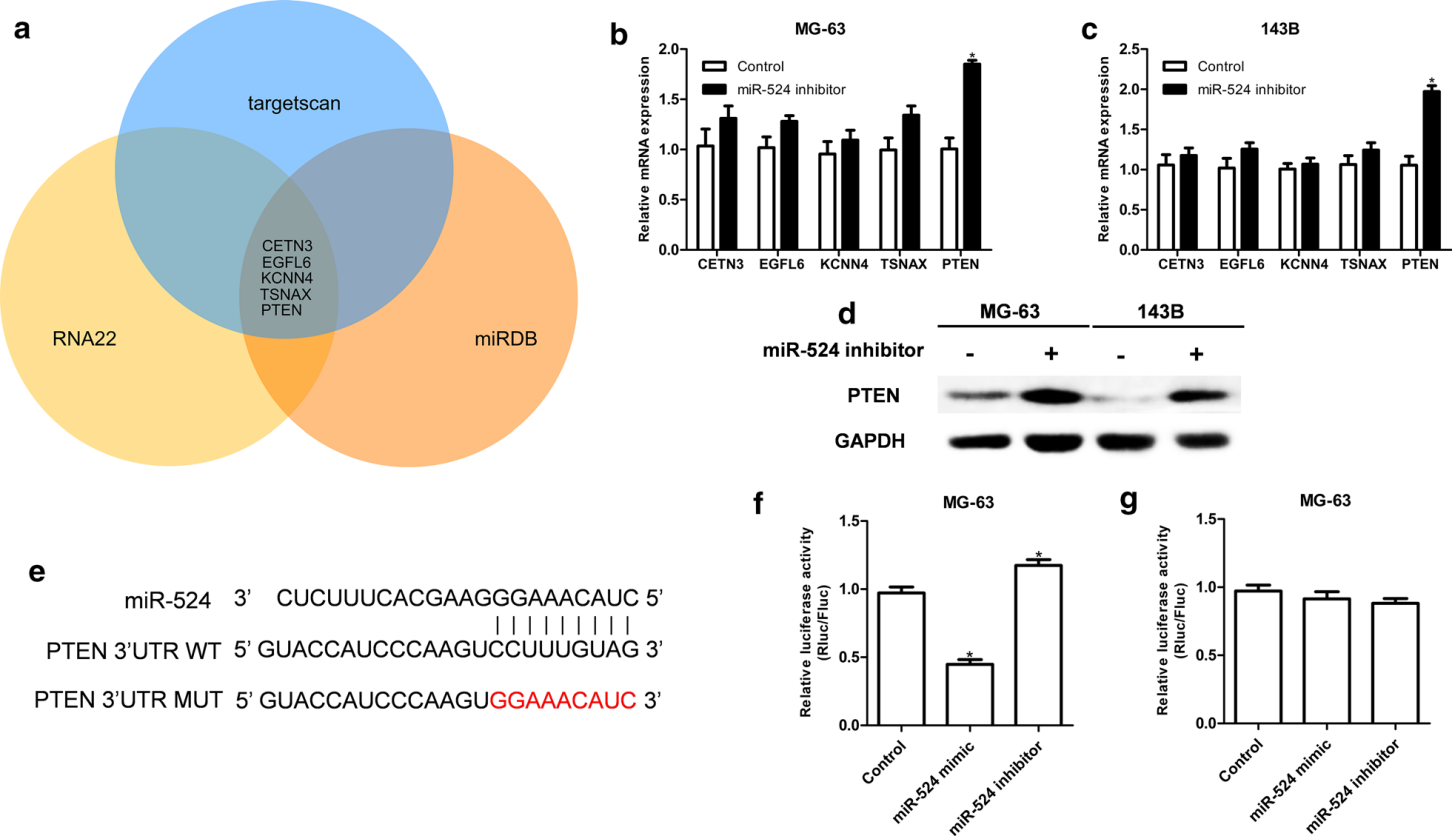
**a** TargetScan, miRDB and RNA22 are used to search for potential target genes of miR-524. After down-regulation of miR-524 expression in MG-63 (**b**) and 143B (**c**) cell lines, mRNA expression levels of each candidate target gene is analysed by qRT-PCR. **d** After down-regulation of miR-524 in MG-63 and 143B, PTEN protein expression was significantly increased. **e** miR-524 and PTEN 3′UTR binding sequences and PTEN 3′UTR mutant sequences. **f**, **g** Luciferase reporter assay used to verify direct binding between miR-524 and PTEN (***P < 0.001, **P < 0.01, *P < 0.05)

**Fig. 4:**
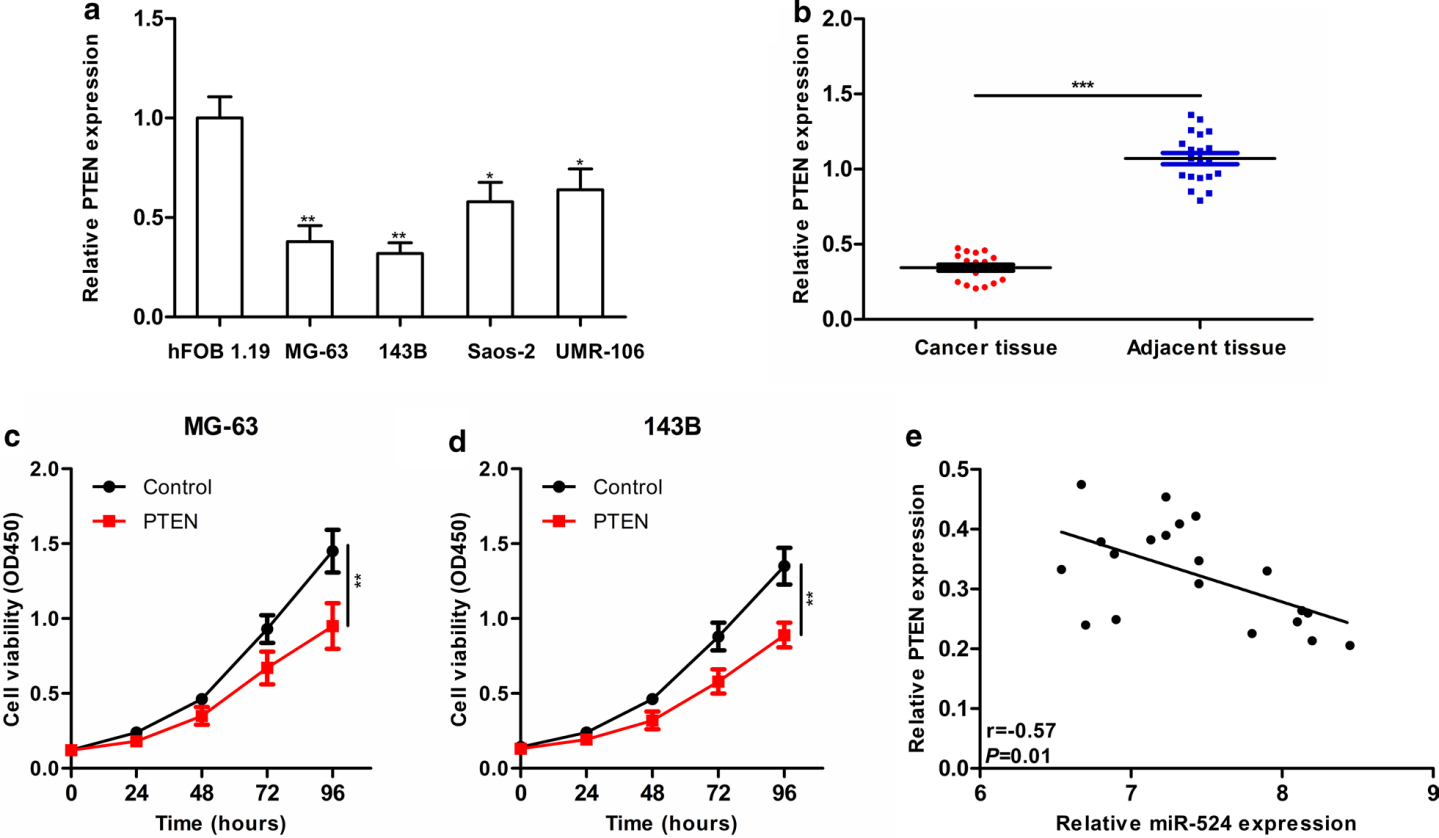
**a** Detection of differences in PTEN expression in normal osteoblasts (hFOB 1.19) and osteosarcoma cell lines (MG-63, 143B, Saos-2 and UMR-106). **b** Detection of differences in PTEN expression between osteosarcoma and para-carcinoma tissues by qRT-PCR. After overexpression of PTEN in MG-63 (**c**) and 143B cells (**d**), proliferation is significantly decreased. **e** There is a negative correlation between miR-524 and PTEN expression levels in osteosarcoma tissues (n = 20) (***P < 0.001, **P < 0.01, *P < 0.05)

To search for potential target genes of miR-524, target genes (CETN3, EGFL6, KCNN4, TSNAX and PTEN) were predicted via TargetScan (http://www.targetscan.org/vert_72/), miRDB (http://www.mirdb.org/) and RNA22 (https://cm.jefferson.edu/rna22/Precomputed/) (Fig. 3a). We next down-regulated the expression of miR-524 in MG-63 and 143B cell lines to determine whether expression of candidate target genes was altered in response. We found that after expression of miR-524 was down-regulated, PTEN mRNA expression was significantly increased, while there were no changes in the expression levels of other candidate target genes (Fig. 3b, c). Furthermore, we found that PTEN protein expression was significantly increased after down-regulation of miR-524 in both MG-63 and 143B cells (Fig. 3d). These results indicate that PTEN is a possible target gene of miR-107. PTEN 3′-UTR wild-type and mutant sequences were then cloned into the luciferase reporter plasmid psiCheck2 and co-transfected into MG-63 cells with either the miR-524 mimic or inhibitor (Fig. 3e). Our results revealed that co-transfection with wild-type PTEN 3′UTR fluorescent plasmid and miR-524 significantly altered the fluorescence intensity (Fig. 3f), and co-transfection with mutant PTEN 3′UTR fluorescent plasmid and miR-524 did not change the fluorescence intensity (Fig. 3g), illustrating that PTEN is a direct target gene of miR-524. PTEN acts as a tumour-suppressor gene in osteosarcoma, which has been confirmed in multiple studies. We detected expression of PTEN in both osteosarcoma cell lines and tissues, finding that PTEN expression was reduced in these samples (Fig. 4a, b) and that cellular function was altered in response to up-regulation of PTEN (Fig. 4c, d). The correlation between miR-524 and PTEN was further analysed, and it was found that PTEN levels were negatively correlated with expression of miR-524 in tissues (Fig. 4e).

### MiR-524 enhances proliferation of osteosarcoma cells through activation of PI3K/Akt signalling via targeting of PTEN

**Fig. 5:**
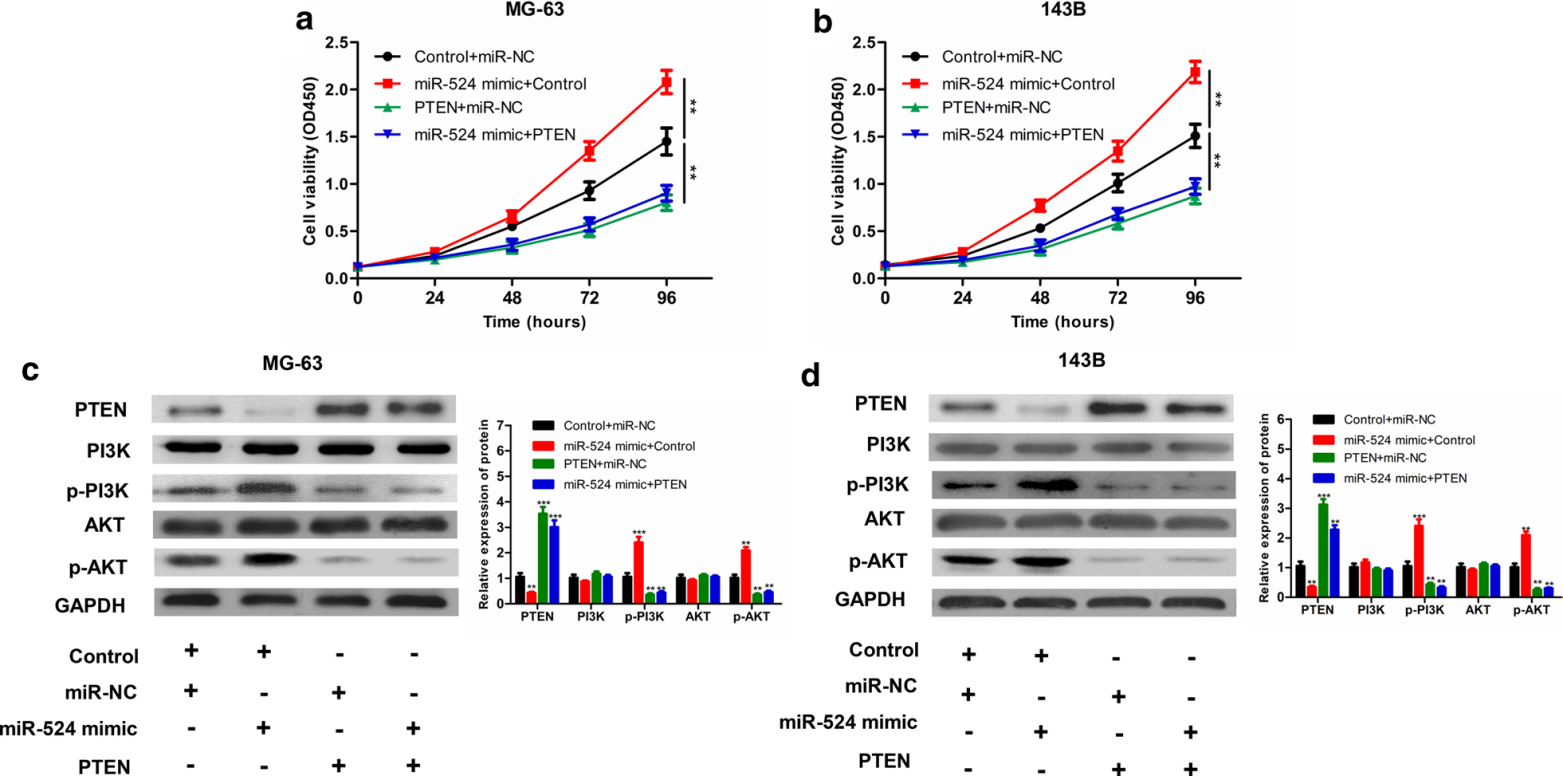
MG-63 (**a**) and 143 (**b**) cells are transfected with Control + miR-NC, miR-524 mimic + Control, PTEN + miR-NC or miR-524 mimic + PTEN, and cell proliferation in each group is detected by CCK-8. The protein expression of PTEN, PI3K, p-PI3K, AKT and p-AKT in MG-63 (**c**) and 143B (**d**) with different treatments: Control + miR-NC, miR-524 mimic + Control, PTEN + miR-NC or miR-524 mimic + PTEN (***P < 0.001, **P < 0.01, *P < 0.05)

To clarify whether PTEN is a functional target gene of miR-524, we performed a rescue experiment. A PTEN overexpression plasmid without 3′UTR was transfected into osteosarcoma cell lines MG-63 and 143B with stable overexpression of miR-524 to investigate whether PTEN could reverse the functional changes in osteosarcoma cells caused by miR-524. Cell proliferation assays revealed that after transfection of PTEN without 3′UTR into osteosarcoma cells with overexpression of miR-524, proliferation capacity was reduced compared with that of the control group, indicating that PTEN effectively reverses miR-524-induced enhancement of proliferation (Fig. 5a, b).

**Fig. 6:**
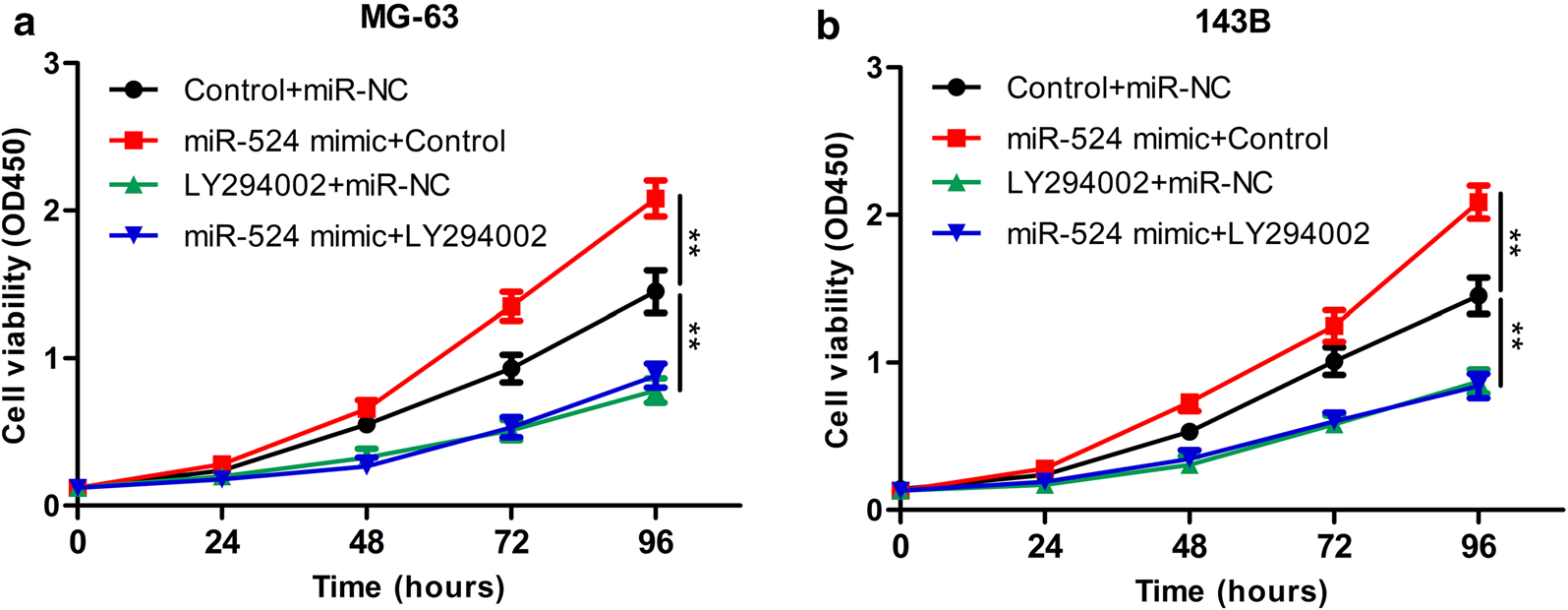
MG-63 (**a**) and 143B (**b**) cells are transfected with Control + miR-NC, miR-524 mimic + Control, LY294002 + miR-NC or miR-524 mimic + LY294002, and cell proliferation in each group is detected by CCK-8

Inhibiting expression of PTEN can up-regulate phosphorylation of AKT. Therefore, we examined whether miR-524 enhances proliferation of osteosarcoma cells through targeting PTEN and further activating the PI3K/AKT signalling pathway. First, we examined the expression of PTEN and its downstream target genes (PI3K, p-PI3K, AKT, p-AKT) in osteosarcoma cells transfected with miR-524 or PTEN or both. Western blot analysis indicated that overexpression of miR-524 can promote the up-regulation of PTEN and down-regulation of p-PI3K and p-AKT in MG-63 and 143B (Fig. 5c, d). Overexpression of PTEN also down-regulated expression of p-PI3K and p-AKT in MG-63 and 143B (Fig. 5c, d). Second, we blocked this pathway using the PI3K/AKT pathway inhibitor LY294002 to observe whether proliferative capacity was changed. The PI3K/AKT inhibitor LY294002 was added to the osteosarcoma cell lines MG-63 and 143B with overexpression of miR-524, and we found that in response to LY294002, cell proliferation induced by miR-524 was completed reversed (Fig. 6a, b). The above experiments prove that miR-524 induces osteosarcoma cell proliferation through activation of the PI3K/AKT pathway via inhibition of PTEN.

## Discussion

More than 2500 mature miRNAs have been identified in the human body thus far. A large number of studies have confirmed that miRNAs are involved in regulating a variety of biological processes, including embryogenesis, organ development, cell proliferation, differentiation, migration and invasion. Dysregulation of miRNA expression can cause a variety of human diseases, including tumours. Recent studies have found that abnormal miRNA expression is closely associated with progression of various malignant tumours, such as gastric cancer [13], liver cancer [14], lung cancer [15] and osteosarcoma [16]. In this study, we found that the miRNA with the most significant differential expression in osteosarcoma was miR-524, which was significantly up-regulated in both osteosarcoma tissues and cell lines. Knockdown of miR-524 inhibited proliferation and promoted apoptosis of osteosarcoma cells, while overexpression of miR-524 promoted proliferation of osteosarcoma cells. The above findings indicate that miR-524 plays an oncogenic role in osteosarcoma. MiR-524 is a member of C19MC, one of the largest miRNA gene clusters in the human genome, and contains 46 highly homologous miRNAs within a 100-kb genomic region [17]. There are few studies on miR-524 in tumours. However, Nguyen et al. found that overexpression of miR-524 promotes the proliferative capacity of embryonic stem cells [18], consistent with the results in this study.

MiRNA is primarily involved in the formation of the RNA silencing complex through incomplete base pairing with the 3′-UTR of target genes, leading to degradation or inhibition of protein translation of target genes. Therefore, the target genes of miRNA are key to the function of miRNA in cells. In this study, bioinformatics analysis and luciferase assay confirmed that PTEN is a direct target gene of miR-524 and that miR-524 induces proliferation of osteosarcoma cells through activation of the PI3K/AKT pathway via inhibition of PTEN. The PTEN gene is located on human chromosome 10q23.3 and is the only tumour-suppressor gene discovered with phosphatase activity in vivo. Moreover, the PTEN gene is widely expressed in normal human tissue cells. As a new tumour suppressor gene, PTEN has been valued by researchers since its emergence in 1997 and is regarded as the most important tumour-suppressor gene after p53. The most important substrate of PTEN is PIP3, which is the product of PI3K and mediates activation of AKT. PTEN dephosphorylates PIP3 to maintain low levels of PIP3, thereby down-regulating the PI3K/AKT pathway. Inactivation of PTEN inevitably leads to the activation of PI3K/AKT. Activated AKT has a variety of biological functions that promote growth and proliferation of tumour cells, inhibit apoptosis, promote invasion and metastasis, and regulate endothelial growth and angiogenesis via catalysing a series of protein phosphorylation reactions [19]. Deletion of the PTEN gene and resulting phosphorylation of AKT are important mechanisms involved in the occurrence of haematologic malignancies. After drug-resistant L cell lines were transfected with PTEN, the sensitivity of these cells to arsenious acid [20] and doxorubicin [21] increased. Moreover, PTEN plays an important role in multi-drug resistance mediated by PI3K/AKT-related signal transduction pathways. Selective PI3K/AKT/NF-kB inhibition or high expression of PTEN can reverse drug resistance in leukaemia. Abnormal expression of PTEN leads to activation of the PI3K/AKT pathway, which up-regulates expression of mTOR, promotes proliferation of tumour cells, inhibits cell apoptosis and mediates multi-drug resistance of cells through PTEN/PI3K/AKT/mTOR [22]. The PI3K/AKT signalling pathway also plays an important role in the migration of cancer cells. The PI3K/AKT signalling pathway promotes cancer cell invasion through up-regulation of MMP2 via multiple pathways [23]. Previous studies have demonstrated that the activation of the PI3K/AKT pathway due to the deletion of PTEN enhances migration and invasion of tumour cells, while the overexpression of PTEN inhibits cell migration. In addition, PTEN not only inhibits cell migration and invasion through inhibition of the PI3K/AKT pathway but also negatively regulates transduction of FAK/P13CAS, as well as integrin-associated kinase signalling through protein phosphatase activity, which plays an inhibitory role in integrin-mediated cell adhesion, migration and invasion. Currently, several studies have proven that PTEN expression is reduced in osteosarcoma and plays a role as a tumour-suppressor gene in osteosarcoma [24–28]. In this study, PTEN inhibits proliferation of osteosarcoma cells, and the mechanism whereby this occurs includes inhibition of the PI3K/AKT pathway.

## Conclusion

The value of miR-524 in osteosarcoma was confirmed in this study for the first time, and we demonstrated that miR-524 induces proliferation of osteosarcoma cells through activation of the PI3K/AKT pathway via inhibition of the target gene PTEN. These findings provide a theoretical basis for selecting a new therapeutic target for osteosarcoma.

## Additional file

**Additional file 1: Table S1.** Primer sequences used for qRT-PCR assays.
