# Supplementary material for: MicroRNA-524 promotes cell proliferation by down-regulating PTEN expression in osteosarcoma
Source: Cancer Cell Int. 2018 Aug 13;18:114. doi: 10.1186/s12935-018-0612-1 (PMC6090628; doi:10.1186/s12935-018-0612-1)
Supplement: Supplementary file 1 — Additional file 1: Table S1. Primer sequences used for qRT-PCR assays. [file 12935_2018_612_MOESM1_ESM.docx]

| Gene | Sequence of the primers |
| --- | --- |
| PTEN-Forward | 5'-TGGATTCGACTTAGACTTGACCT-3' |
| PTEN-Reverse | 5'-GGTGGGTTATGGTCTTCAAAAGG-3' |
| CETN3-Forward | 5'-TGGCAATGAGAGCCTTGGG-3' |
| CETN3-Reverse | 5'-AACACGTCGCAAATTCCTCAA-3' |
| EGFL6-Forward | 5'-AGGCATCACGGGTTGTTAGC-3' |
| EGFL6-Reverse | 5'-CGTAGCAGCAGGCCAGTTTAG-3' |
| KCNN4-Forward | 5'-TTGGCTGATCCCCATCACATT-3' |
| KCNN4-Reverse | 5'-CAGGCTTCTTGTAGCACTCGG-3' |
| TSNAX-Forward | 5'-GACTGAGAGTCACACCTGTCG-3' |
| TSNAX-Reverse | 5'-CCTCGTAAGGTCCAGTGTTGC-3' |
| GAPDH-Forward | 5'-CTCACCGGATGCACCAATGTT-3' |
| GAPDH-Reverse | 5'-CGCGTTGCTCACAATGTTCAT-3' |
| miR-524 | 5'-CUCUUUCACGAAGGGAAACAUC-3' |
| U6 | 5'-CGCAAGGATGACACGCAAATTC-3' |

Additional file 1: Table S1 Primer sequences used for qRT-PCR assays
